# Identification of *IPT9* in *Brachiaria brizantha* (s*yn. Urochloa brizantha*) and expression analyses during ovule development in sexual and apomictic plants

**DOI:** 10.1007/s11033-023-08295-7

**Published:** 2023-04-18

**Authors:** Luciana G. Ferreira, Diva M. A. Dusi, André S. T. Irsigler, Ana C. M. M. Gomes, Lilian H. Florentino, Marta A. Mendes, Lucia Colombo, Vera T. C. Carneiro

**Affiliations:** 1 Department of Biology, University of Brasília - UnB, Campus Darcy Ribeiro S/N – Asa Norte, Brasília, DF 70.910-900 Brazil; 2 Embrapa Genetic Resources and Biotechnology, Parque Estação Biológica, PqEB Av. W5 Norte., Caixa Postal 02372, Brasília, DF 70.770-917 Brazil; 3 Dipartimento di Bioscienze, Università degli Studi di Milano, Via Celoria 26, 20133 Milan, Italy

**Keywords:** Apomixis, Apospory, Megagametogenesis, Megasporogenesis, Reproduction

## Abstract

**Background:**

In *Brachiaria* sexual reproduction, during ovule development, a nucellar cell differentiates into a megaspore mother cell (MMC) that, through meiosis and mitosis, gives rise to a reduced embryo sac. In aposporic apomictic *Brachiaria*, next to the MMC, other nucellar cells differentiate into aposporic initials that enter mitosis directly forming an unreduced embryo sac. The *IPT* (isopentenyltransferase) family comprises key genes in the cytokinin (CK) pathway which are expressed in Arabidopsis during ovule development. *BbrizIPT9*, a *B. brizantha* (*syn. Urochloa brizantha*) *IPT9* gene, highly similar to genes of other Poaceae plants, also shows similarity with Arabidopsis *IPT9, AtIPT9.* In this work, we aimed to investigate association of *BbrizIPT9* with ovule development in sexual and apomictic plants.

**Methods and results:**

RT-qPCR showed higher *BbrizIPT9* expression in the ovaries of sexual than in the apomictic *B. brizantha*. Results of in-situ hybridization showed strong signal of *BbrizIPT9* in the MMC of both plants, at the onset of megasporogenesis. By analyzing *AtIPT9* knockdown mutants, we verified enlarged nucellar cell, next to the MMC, in a percentage significantly higher than in the wild type, suggesting that knockout of *AtIPT9* gene triggered the differentiation of extra MMC-like cells.

**Conclusions:**

Our results indicate that *AtIPT9* might be involved in the proper differentiation of a single MMC during ovule development. The expression of a *BbrizIPT9*, localized in male and female sporocytes, and lower in apomicts than in sexuals, and effect of *IPT9* knockout in Arabidopsis, suggest involvement of *IPT9* in early ovule development.

**Supplementary Information:**

The online version contains supplementary material available at 10.1007/s11033-023-08295-7.

## Introduction

*Brachiaria*, a genus of the Poaceae family, has the most cultivated and economically important species reproducing by apomixis, asexual reproduction through seeds [1]. Female reproductive pathway in *Brachiaria* sexual plants, as in many other angiosperms, comprises three processes inside the ovule: 1-megasporogenesis with the differentiation of a subepidermal cell in the nucellus, the archespore, followed by differentiation of the megaspore mother cell, MMC, which after meiosis results in four reduced megaspores, only one survives as a functional megaspore; 2-megagametogenesis, in which the functional megaspore enlarges and, after three mitosis and cellularization form a reduced seven celled embryo sac (ES) of the Polygonum-type; and 3-double fertilization in which the egg cell and the central cell are fertilized by the sperm cells to form the embryo and endosperm respectively. In *Brachiaria*, apomixis is by apospory. In these plants, ovule development differs from the sexual plants in three moments, before anthesis. At megasporogenesis meiosis may or may not be complete, and the formed tetrad of megaspores then degenerates, or the meiocyte degenerates before dyads or tetrads can be formed [2, 3]. Meanwhile, a number of nucellar cells differentiate to form the aposporic initials, cells capable of developing ES without meiosis. At megagametogenesis, the aposporic intials then divide by mitosis and form the unreduced four cellular ES of the Panicum-type, in which the embryo will develop autonomously from the egg cell, but fertilization of the central cell is still required to form the endosperm [2–4]. Occasionally, a meiotic ES can be observed in apomictic plants, a feature that led to the conclusion that apomixis in *Brachiaria* is facultative [5]. Because apomixis leads to the production of clonal seeds, and there is a possibility of genetic control of the trait, it gained the attention of the international scientific community working with plant reproduction and breeding in different species [6–12]. Identification of genes related to apomixis may, in one hand, allow the transfer of apomixis to important crops, fixing superior genotypes maintaining hybrid vigor, and on the other hand, the turn off of apomixis in natural apomictic plants such as *Brachiaria*, simplifying breeding. To discover genes associated with the main steps of ES differentiation, we have been analyzing gene expression in ovules of sexual and apomictic *Brachiaria brizantha* (syn. Urochloa *brizantha*) plants [13–18]. In a previous comparison of RNA Seq libraries from ovaries of sexual and apomictic *B. brizantha*, a gene with characteristics of isopentenyltransferases (IPTs) was selected due to the substantial difference in the relative expression profile between sexual and apomictic *B. brizantha*. IPTs mediate the biosynthesis of cytokinins (CKs) in different organisms [19, 20], and are expressed during all phases of ovule development in Arabidopsis [21, 22].

In higher plants, IPTs catalyze the initial step of de novo biosynthesis of CK [23]. ATP/ADP IPTs are responsible for isopentenyladenine- and trans-zeatin-type CK synthesis, the most of the CK synthesis, while tRNA IPTs are required for cis-zeatin-type CK production [20]. In Arabidopsis seven ATP/ADP IPTs, AtIPT1, AtIPT3, AtIPT4, AtIPT5, AtIPT6, AtIPT7, AtIPT8 [24] and two tRNA IPTs, AtIPT2 and AtIPT9 were reported [25, 26]. The tRNA IPTs show constitutive expression, with stronger intensity in roots, shoot apical meristems and leaf primordia [27]. In *Jatropha curcas*, the upregulation of IPT and downregulation of CK dehydrogenase, which catalyzes CK degradation, during the stage of MMC occurrence, suggest the enhancement of CK biosynthesis at this stage [28]. During Arabidopsis ovule development, CK biosynthesis was detected by pIPT1::GUS which was expressed at MMC and functional megaspore stages, specifically at the nucellus and at the mature ovule in the ES [21].

The plant hormone CK was already known to regulate many aspects of plant growth and development, such as cell division, apical dominance, nutrient mobilization, seed germination, leaf expansion, and delay of senescence [29–31]. These hormones act during reproductive development, and in Arabidopsis have been associated with the activity of reproductive meristems, flower organ size, ovule formation, seed yield [22, 32], functional megaspore specification [33], regulation of ovule development and correct determination of ovule number [21, 34, 35]. It has been suggested that CK may regulate the occurrence of MMC [28]. Although the influence of CK levels in ovule pattern has been shown in Arabidopsis [21]and null mutants for the ATP/ADP IPT and tRNA IPT were isolated [26], the role of IPTs in Arabidopsis ovule development is still not described. We present in this manuscript the *B. brizantha IPT9, BbrizIPT9,* its expression associated with ovule and ES development in sexual and apomictic *B. brizantha*. In addition, the effect of *AtIPT9* knockout during ovule development in Arabidopsis is presented.

## Materials and methods

### Plant material

A sexual diploid (2n = 2x = 18) accession, BRA 002,747, and a facultative apomictic tetraploid (2n = 4x = 36) accession, BRA 000,591, named *B. brizantha* cv. Marandu [5], were used in this work. Pistils, ovaries and anthers were dissected from hermaphrodite flowers before anthesis at different stages of development. Stages I and II contain ovules in megasporogenesis, while stages III and IV contain ovules in megagametogenesis. At stage I, pistils have very short stigmas and ovules with nucellar cells and MMC; at stage II, pistils have elongated stigmas and ovules with megaspores and, only in the apomictic, aposporic initials; at stage III, pistils have white hairy stigma, and in sexual ovule, one immature meiotic ES is observed, while in the apomictic ovule multiple immature aposporic ES are present; at stage IV, long pistils have red hairy stigmas and ovule with mature ES of Panicum-type, in apomictic plants and Polygonum-type in sexual plants [2, 17].

The Arabidopsis *ipt9* mutants (SALK_027711 and SALK_128927), with insertions at the *AtIPT9* 5′UTR, developed at the Salk Institute-USA, were ordered from the Arabidopsis Biological Resource Center (ABRC). Mutants and wild-type Arabidopsis (ecotype Columbia) were cultivated at 22 °C under 16/8 h light/dark schedule, and genotyped by PCR, using the primers 5′ GCTACGCCACATAAAGGAAT 3′ and 3′ ACGGTTCGGTTAGGTTTACA 5′, for amplification of a 765 bp fragment from a wild-type plant. To verify if the plant mutants were homozygous, the primer 3′ATTTTGCCGATTTCGGAAC 5′ for the amplification of T-DNA together with the primer 5′ GCTACGCCACATAAAGGAAT 3′ were used to detect amplification of a mutant band of 350 bp. The heterozygotes display two fragments. In the SALK_027711 mutant, the relative expression of *AtIPT9* was verified by RT-qPCR.

### Sequence analysis

A *B. brizantha* gene sequence of 866 nucleotides was previously identified as differentially expressed in RNA-Seq libraries of sexual and apomictic ovaries of *B. brizantha*. This sequence was used to screen genomic libraries generated from short Illumina reads of sexual and apomictic *B. brizantha* in order to rescue the full-length. Nucleotides and predicted amino acid sequences of sexual and apomictic *B. brizantha* plants were submitted to *The Basic Local Alignment Search Tool* (BLAST) [36, 37] performed against the *National Center for Biotechnology Information* (NCBI; http://www.ncbi.nlm.nih.gov). The NCBI *Conserved Domain Search* was used to identify a common conserved sequence with the tRNA delta (2)-*isopentenyl pyrophosphate transferase* (IPP transferase) family. Partial sequences of nucleotide and amino acid sequence were used in multiple sequence alignments with ClustalW2 [38] and homologs were defined with a cutoff *e*-value of ≥ 2e^− 57^ and identity above 90%. The sequence was compared to Arabidopsis sequences obtained from The Arabidopsis Information Resource database (TAIR, http://arabidopsis.org). The genomic sequences from sexual and apomictic genotypes were identified in genomic libraries and analyzed with Webcutter 2.0. gene. A phylogenetic tree based on full protein sequences of the isopentenyltransferase (IPT) family in Arabidopsis and a IPT from *B. brizantha* was constructed using the Maximum Likelihood-based method with PhyML v20160115 [39], based on 100 bootstrapped trees. Abbreviations: AT, *Arabidopsis thaliana*; B105, *B. brizantha* sexual.

### RNA isolation and RT-qPCR analysis of *B. brizantha* plants

Total RNA was extracted from sexual and apomictic *B. brizantha* plants, in three biological replicates, each one consisted of a pool of 250 ovaries collected in different plants, at each of the four stages [2], using TRIZOL® (Invitrogen) as previously described [13, 17]. The reverse transcriptase reaction was performed with SuperScript III (Invitrogen) and 2 µg of RNA, according to the manufacturer’s protocol. RT-qPCR was performed using SYBR Green PCR Master Mix Green (Applied Biosystems). Oligonucleotide pairs for *BbrizIPT9,* designed with Primer 3.0 program [40], were: 5′ GAGATCATCAGCGCCGACTC and 3′ GGACAGGGACTGGTACTGGAAG 5′. ΔΔCt analysis was done using *BbrizUBCE* as reference gene, encoding a ubiquitin conjugating enzyme, amplified with 5′ GGTCTTGCTCTCCATCTGCT 3′ and 3′ CGGGCTGTCGTCTCATACTT 5′ [41]. Three technical replicates of each of the three different biological replicates were used.

### RNA isolation and RT-qPCR analysis of Arabidopsis plants

Total RNA of Arabidopsis cv. Columbia and SALK_027711 was extracted from 1–2 inflorescences in three biological replicates with NucleoZOL® (Macherey–Nagel) according to the manufacturer’s instructions. After incubation with the Ambion TURBO DNA-free DNase (Invitrogen), RNA was reverse transcribed using the ImProm-II™ reverse transcription system (Promega).

The oligonucleotide pairs, designed with Primer 3.0 program, were for *AtIPT9* 5′TCAATGCTGGTGATCCAAAA3′ and 5′TCATCAGCATCAGGAGCAAC3′, *UBIQUITIN10* 5′CTGTTCACGGAACCCAATTC3′ and 5′GGAAAAAGGTCTGACCGACA3′, and *ACTIN2-8* 5′CTCAGGTATTGCAGACCGTATGAG3′ and 5′CTGGACCTGCTTCATCATACTCTG 3′. *UBIQUITIN10* and *ACTIN2-8* genes were used as references, and gene expression analysis was performed with iQ5 Multicolor real-time PCR detection system (Bio-Rad) with SYBR Green PCR Master Mix (Bio- Rad). Three technical replicates of each of the three different biological replicates were used.

### In situ hybridization analysis

Semi-thin Sects. (3.5 μm) of ovaries at megasporogenesis of sexual and apomictic *B. brizantha* were hybridized with an 866 bp PCR fragment of *BbrizIPT9*, previously cloned into pGEM-T Easy Vector System I (Invitrogen) and used as template for sense and antisense probes, with SP6 and T7 polymerases respectively. A DIG RNA labeling kit (Roche) was used according to the manufacturer’s protocol. The sample preparation and in situ hybridization procedures were carried out as previously described [13, 17, 18, 42]. The hybridized section slides were observed using a Zeiss Axiophot light microscope.

### Morphological analysis of Arabidopsis flowers by DIC microscopy

Flowers were fixed with 9:1 ethanol:acetic acid and cleared overnight using a clearing solution of chloral hydrate:glycerol:water (3:1:2; w/v/v). Ovules were dissected from premature pistils after clearing, mounted with a cover slip, and subsequently observed in a Zeiss Axiophot D1 microscope equipped with DIC optics. Images were captured on an Axiocam MRc5 camera (Zeiss) using the Axiovision program (version 4.1). Three different plants of Arabidopsis ecotype Columbia and of each mutant, SALK_027711 and SALK_128927, were analyzed.

## Results

### *BbrizIPT9* has sequence similarity to *IPT9 *gene

**Fig. 1:**
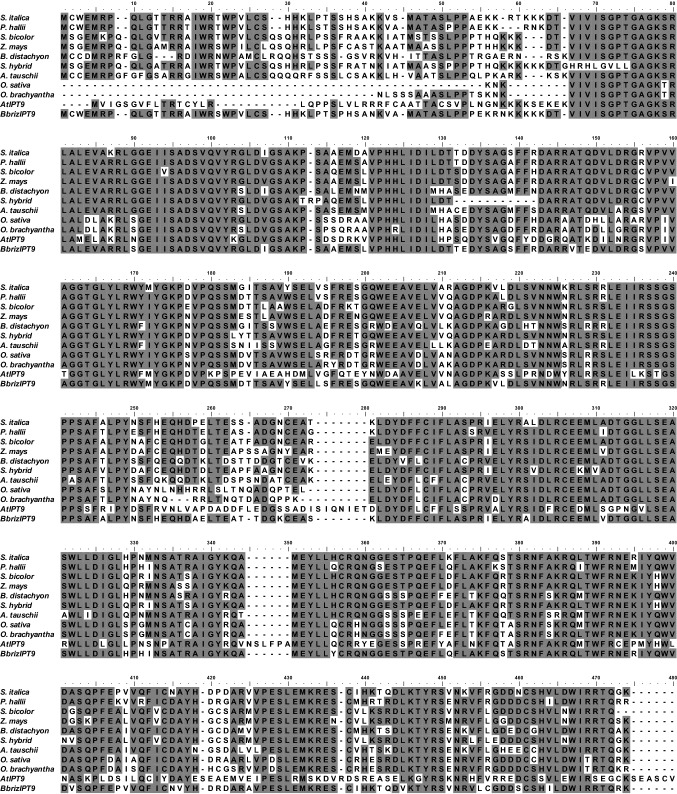
Multiple alignment of *BbrizIPT9* and similar sequences found in the NCBI database: *Setaria italica* (XP_004963310.1), *Panicum hallii* (XP_025807403.1), *Sorghum bicolor* (XP_002463348.1), *Zea mays* (AQK80671.1), *Brachypodium distachyon* (XP_003563223.2), *Saccharum* hybrid cultivar R570 (AGT16524.1), *Aegilops tauschii* (XP_020163508.1), *Oryza sativa* japonica group (XP_015644187.1), *Oryza brachyantha* (XP_006656524.1), and *Arabidopsis thaliana* (AT5G20040). Gray highlight shows the amino acid residues conserved amongst the sequences. Multiple sequence alignment was performed using the ClustalW program

Previous RNA-Seq analysis pointed to a sequence of 866 nucleotides from sexual and apomictic *B. brizantha*, which was similar to the *isopentenyltransferase 9* family from different plants. Screening of genomic libraries of sexual and apomictic *B. brizantha* revealed a full-length DNA sequence of 1356 nucleotides, in sexual and apomictic plants (Fig. S1). The sequence, without introns detected, encodes 451 amino acids (Fig. S2) and shares a common conserved sequence with the IPP transferase family, as indicated by The NCBI *Conserved Domain Search*. *B. brizantha* amino acid derived sequence shows high similarity with IPT9 sequences from other species of the Poaceae family, in different percentages, as verified using BLAST in the NCBI database (Fig. 1): *Setaria italica* (90%), *Panicum hallii* (87%), *Sorghum bicolor* (80%), *Zea mays* (77%), *Saccharum* hybrid cultivar R570 (75%), *Brachypodium distachyon* (73%), *Oryza sativa* Japonica Group (73%), *Oryza brachyantha* (71%) and *Aegilops tauschii* (70%). Among the members of the Arabidopsis *IPT* sequences*,* the *B. brizantha* sequence showed a higher level of identity with *AtIPT9* (55%) [26] (Fig. S3). Contigs of genomic sequences from the sexual BRA 002,747 (B105) and the apomictic BRA 000,591 (B30) genotypes, with the coding region for the *B. brizantha IPT9* were deposited in the GenBank as MZ724659 and MZ724660.

### *BbrizIPT9* expression differs in ovaries of sexual and apomictic *B. brizantha*

**Fig. 2:**
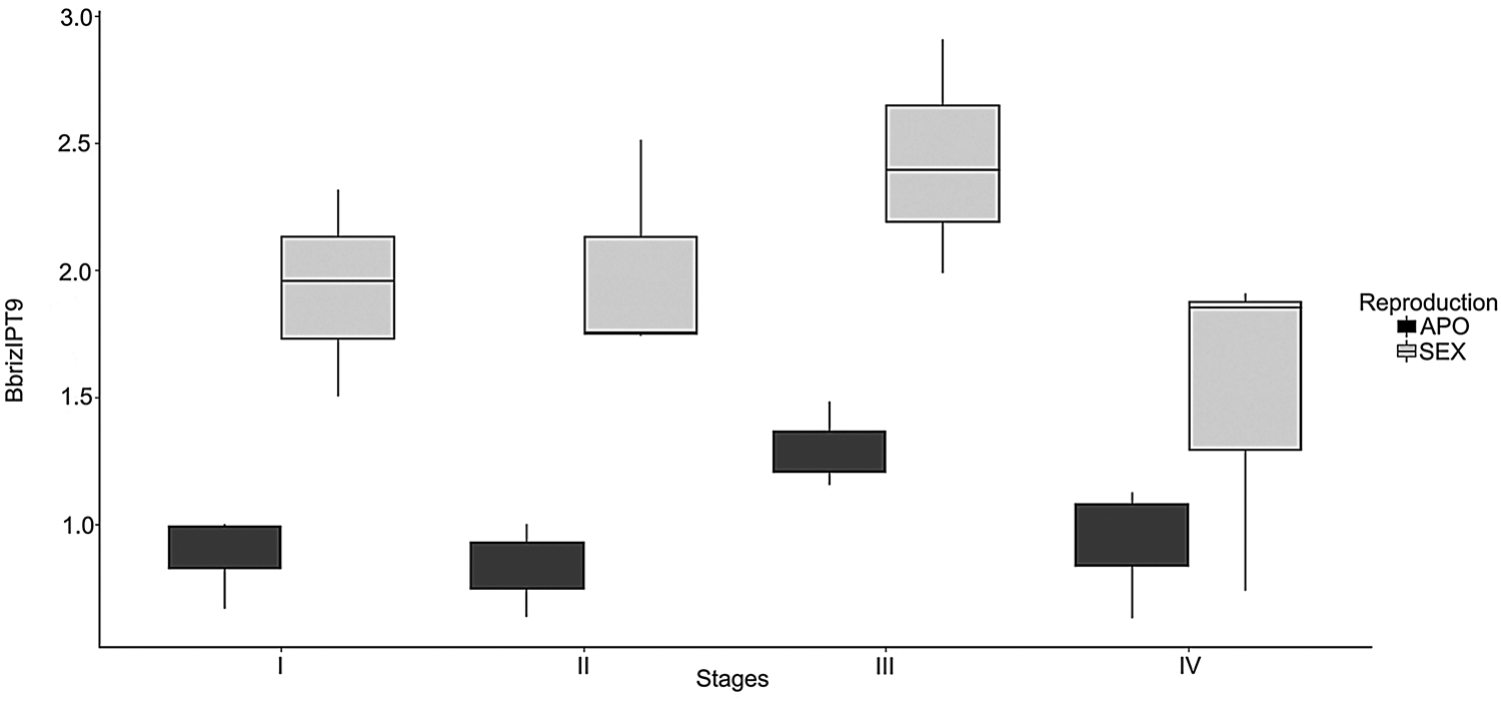
Boxplot diagram showing the *BrizIPT9* relative expression detected by RT-qPCR in developing ovaries of sexual and apomictic *Brachiaria brizantha.* Analysis was performed in ovaries at early and late megasporogenesis (stages I and II respectively) and megagametogenesis (stages III and IV respectively) before anthesis, of apomictic plants (APO) and sexual plants (SEX). Bars represent standard deviations based on the average of three biological samples. The mean quartiles, median and ANODEV were calculated using the R Core Team (2016) program

RT-qPCR validated previous data of RNA-Seq analysis resulting in higher expression of *BbrizIPT9* in ovaries of sexual plant compared to ovaries of the apomictic *B. brizantha. BbrizIPT9* transcript levels were different among sexual and apomictic plants, during ovary development, at three different stages before anthesis (Fig. 2). *BbrizIPT9* expression is higher in sexual than in apomictic plants in megasporogenesis and early stage of megagametogenesis, as shown by the analysis of deviance (ANODEV), with a 1% significance, that displayed a difference between the reproductive modes (F = 43.0960, GL = 1, p = 2,764e^06^), but not among the stages of ovule development (F = 2.8013, GL = 3, p = 0.0678) in either apomicts or sexual plants. This difference was observed in ovules with MMC (stage I), ovules with megaspores, and, only in the apomictic, aposporic initials (stage II), and ovules showing immature meiotic ES or the multiple immature aposporic ES in the case of apomixis (stage III). In mature ES of Polygonum-type, in sexuals and Panicum-type in apomicts at stage IV, no significant difference was observed.

### *BbrizIPT9* in situ expression is similar in ovules and anthers of apomictic and sexual *B. brizantha* at sporogenesis

**Fig. 3:**
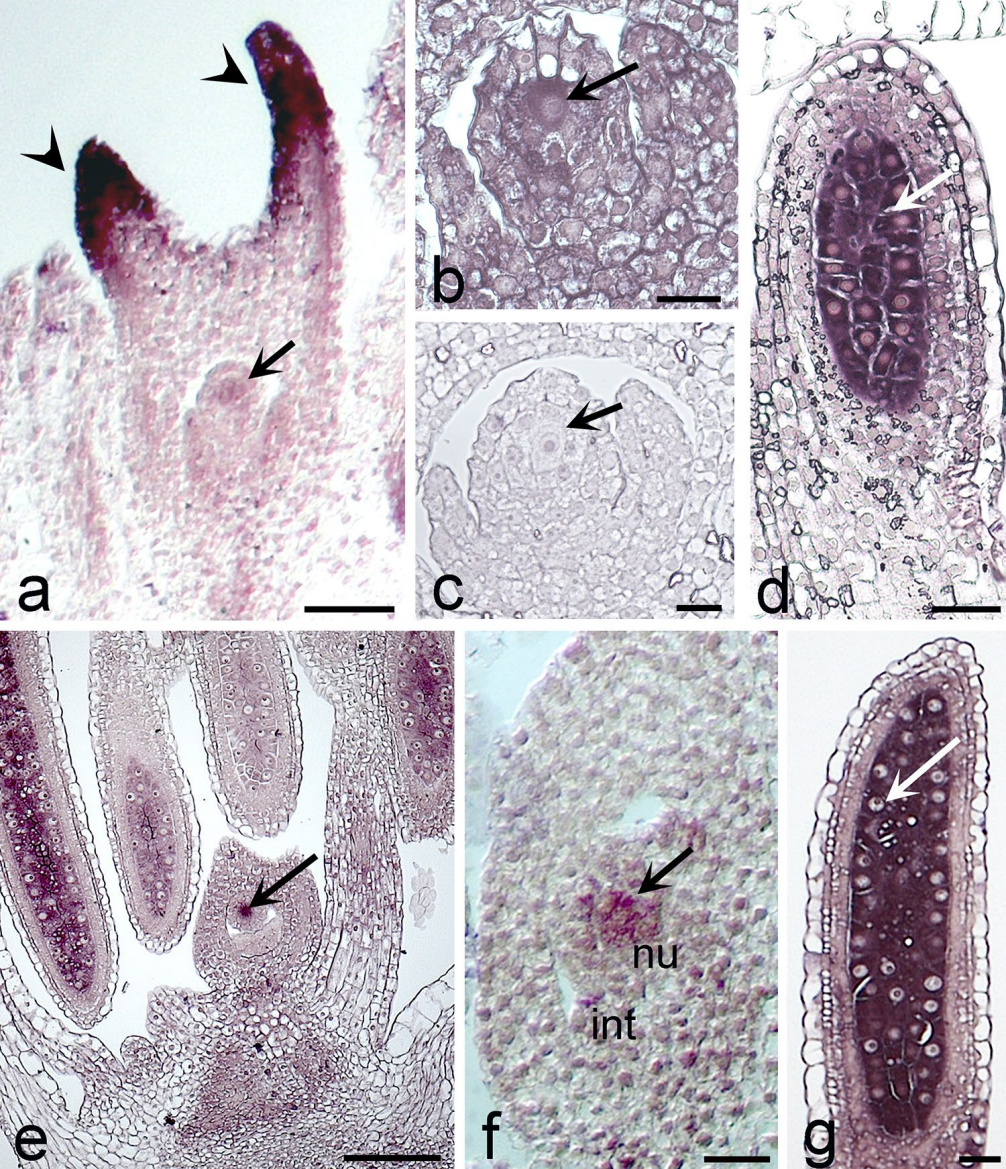
*BbrizIPT9* expression observed by in situ hybridization in apomictic (a-d) and sexual (e–g) plants of *Brachiaria brizantha*. **a** Pistil with weak signal in the archesporic cell (thin arrow) and strong signal in the upper portion of the style (broad arrow). **b** Detail of an ovule showing hybridization signal in the megaspore mother cell, MMC (arrow). **c** Ovary hybridized with sense probe with no signal. **d** Anther with hybridization signal in the pollen mother cells, PMC (arrow). **e** Inflorescence of sexual plant with strong hybridization in MMC (arrow). **f** Ovule detail showing stronger signal of hybridization in the MMC (arrow) than in the surrounding nucellar cells (nu) and weak signal in the integuments (int). **g** Anther of sexual plant with hybridization in the PMC (arrow). Scale bars = a (10 μm), b–d (20 μm), e **(**100 μm), f (20 μm), g (25 μm)

In situ hybridization showed the presence of *BbrizIPT9* transcripts in early stages of ovary development, at megasporogenesis, of sexual and apomictic plants of *B. brizantha* (Fig. 3). In apomictic plants (Fig. 3a-d), a weak hybridization signal was detected in the archespore and a strong signal in the superior portion of the style, the stylodium (Fig. 3a). In *B. brizantha*, the archespore differentiates into the MMC directly without previous division. Expression observed in the ovules was slightly stronger in the MMC than in nucellar cells (Fig. 3b). The sense control probe did not show any signal in the ovaries of apomictic (Fig. 3c) or sexual plants. In anthers, in the pollen mother cells (PMC), a strong hybridization signal was observed (Fig. 3d). In sexual plants (Fig. 3e-g), during megasporogenesis, strong hybridization signal was observed in the MMC and PMC (Fig. 3e and f). Anthers showed strong hybridization signal in the PMC (Fig. 3g).

### Morphological analyses of Arabidopsis *Atipt9* mutant in ovules

**Fig. 4:**
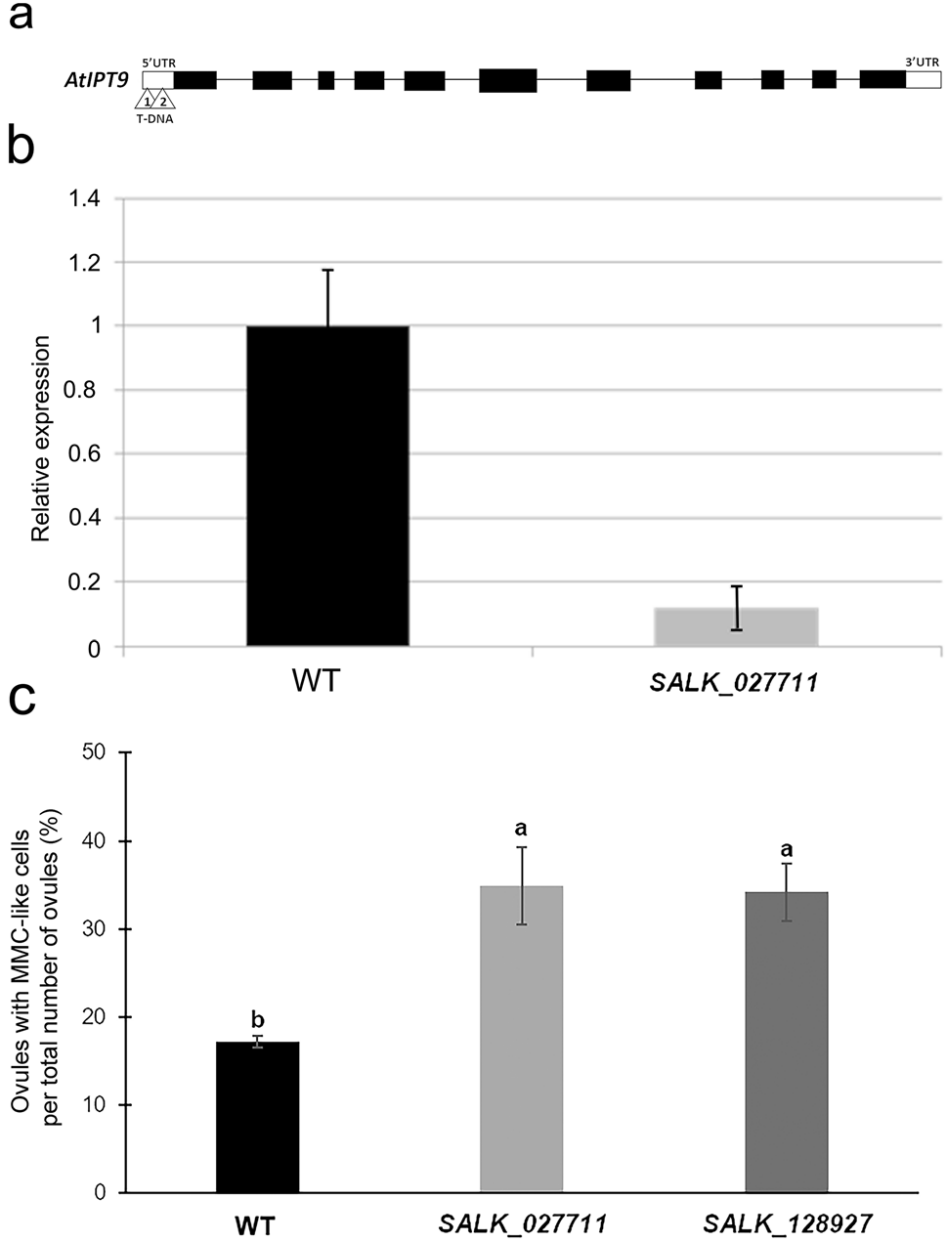
Effect of T- DNA insertions at the *Arabidopsis thaliana AtIPT9* (AT5G20040.3) on MMC-like cells proliferation. **a** Schematic representation of *AtIPT9* genomic structure indicating with arrowheads T-DNA insertion for SALK_128927 (1) and SALK_027711(2) located at different sites of the 5´UTR region. The black blocks represent the exons and the thin lines, the introns. **b** Relative expression levels of the *AtIPT9* in inflorescences of wild-type and *ipt9* mutant (SALK_027711) plants of Arabidopsis by RT-qPCR. The bars represent the standard deviation based on the average of three biological replicates. Actin8 and Ubiquitin10 were used as the reference gene. **c** Percentage of the number of ovules with additional MMC-like cells found in 436 ovules of Arabidopsis wild-type cv. Columbia, 255 ovules of SALK_027711 and 398 ovules of SALK_128927. Different letters indicate statistically significant differences between the percent number of ovules with additional MMC-like cells (p = 0.039). WT: wild-type (Columbia ecotype); MMC = megaspore mother cell

In order to check a putative *IPT9* gene function during ovary development and make comparative studies with *B. brizantha*, Arabidopis *IPT9* knockout mutants were analyzed. The two mutant lines, SALK_027711 and SALK_128927, have T-DNA insertions in a non-coding region of the *IPT9* gene (Fig. 4a). The relative expression of the *IPT9* gene was analyzed in the SALK_027711 line, and the results showed that the expression of *AtIPT9* was close to zero in inflorescences of the mutant plants (Fig. 4b).

Morphological analysis of the ovaries of Arabidopsis *ipt9* knockout mutants SALK_027711 and SALK_128927 containing the T-DNA inserted into the 5′UTR of the gene showed differences from the wild-type plants (Fig. 4c). We have observed at the finger-like stage (2-I), by light microscopy, that the number of ovules with enlarged nucellar cells in proximity and resembling the MMC, herein called MMC-like cells following other authors [13, 43, 44], doubled in both mutants, SALK_027711 and SALK_128927, when compared with the wild-type plants (Fig. 4c).

**Fig. 5:**
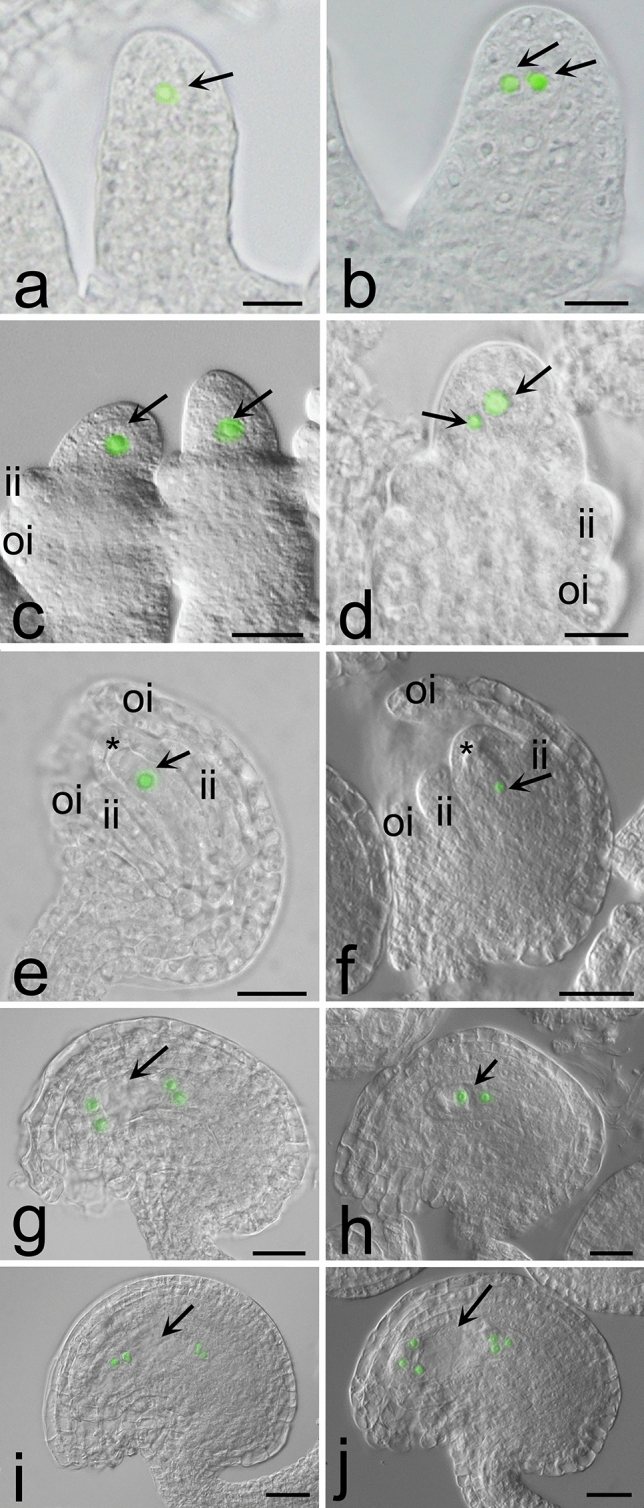
Ovules of wild type (**a**, **c**, **e**, **g**, **i**) and *ipt9* mutant (**b**, **d**, **f**, **h**, **j**) of *Arabidopsis thaliana*, observed with differential phase contrast optical microscopy. **a**—**b** Finger-like stage (2-I and 2-II) showing one MMC (arrow in **a**) and two MMC-like cells (arrows in **b**). **c** – **d** At stage 2-III with the outer and inner integuments differentiating, the MMC-like cells are still visible in the mutant (arrows in **d**). **e**—**f** FG1 with functional megaspore (arrow). **g** FG4 the 4-nucleated developing female gametophyte (arrow). **h** FG3 stage showing the binuclear embryo sac (arrow). **i**, **j** FG6 stage, the arrow indicates the embryo sac. Nuclei were artificially colored in green in the pictures to make them more evident. Asterisks: degenerated megaspores; ii = inner integument; oi = outer integument. Scale bars = a, h (10 μm), b (15 μm), c (25 μm), d-g, i-j = 20 μm

Ovules of wild-type plants followed an expected pattern of development resulting in the formation of only one ES (Fig. 5 a,c,e,g,i). In the *ipt9* mutants (Fig. 5 b,d,f,h,j), in very young ovules, about the 2I-2II stage [45], in addition, MMC-like cells were observed (Fig. 5 b,d). On the FG1 stage (Fig. 5 e, f), like the wild type (Fig. 5e) the mutant had only one functional megaspore (Fig. 5f). After mitosis (Fig. 5 g-j), two-nucleated ES, four-nucleated ES, eight-nucleated ES, and finally a seven-celled ES were observed in both wild type and mutants. In these analyses we have not detected any twin ES. The *ipt9* mutant SALK_027711 and wild-type plants showed similar numbers of seeds per silique. The *ipt9* mutant plants showed an average of 42 seeds per silique, in the 30 siliques counted, with 1.8% aborted seeds. Wild-type plants, under the same environmental conditions, showed an average of 46 seeds per silique in the 32 siliques counted, with a 2.5% abortion rate. Statistical analysis (t-test) showed that there was no significant difference between the number of seeds per silique observed in the *ipt9* mutant and wild-type plants (p = 0.20).

## Discussion

The investigation of the sequence previously detected in the transcriptome as differentially expressed in ovaries of sexual and apomictic *B. brizantha*, herein named *BbrizIPT9*, brought up points to be discussed about reproduction. A remarkable *BbrizIPT9* expression in the MMC of apomictic *B. brizantha*, and especially in the MMC of the sexual *B. brizantha*, suggests a possible relation of *BbrizIPT9* to the differentiation of this cell or to its fate. In apomicts, either the meiocyte or the four megaspores of the tetrad degenerate, while in sexuals, one of the megaspores is functional and enters megagametogenesis. A peak of relative expression at the beginning of megagametogenesis provides evidence of regulation in the course of the reproduction process. In anthers, the PMC from both apomictic and sexual plants also shows high expression. These cells, like the MMC, will enter and complete meiosis to form the spores. In the apomictic plants, the microsporogenesis occurs likewise in sexual plants, resulting in reduced microspores, while in megasporogenesis, the MMC may or may not complete meiosis [2, 3]. Our results indicate a role of *BbrizIPT9* in *B. brizantha* reproductive development, suggesting that *BbrizIPT9* expression is associated with the development of the MMC and PMC, cells that after meiosis, will give rise to megaspores and microspores respectively.

In the Arabidopsis mutants *argonaute* 9 (*ago9*) and *RNA-dependent RNA polymerase* 6 (*rdr6-11*), additional enlarged cells in nucellus, with a conspicuous nucleus and nucleolus were reported [46]. These and other mutants, defective in the RNA-direct DNA methylation (*RdDM*) pathway, seemed to display more than one MMC per ovule. *RdDM* is the major small RNA-mediated epigenetic pathway in plants and it is involved both in transcriptional and post-transcriptional gene silencing. Recent studies [44] demonstrated that *RdDM* and also epigenetic modifications through methylation by DOMAINS REARRANGED METHYLASEs (DRM1 and DRM2) are important for the formation of a single MMC, 65% of *drm1drm2* ovules displayed multiple MMC-like cells. It was verified that only one MMC-like cell acquires MMC identity and undergoes meiosis. In the Arabidopsis *ipt9* knockout mutant described here, only one MMC-like cell undergoes further development to form an ES. *AtIPT9* expression in Arabidopsis inflorescences and the significant increase in the number of MMC-like cells in *ipt9* knockout mutant, suggest that this gene could be part of a mechanism that avoids differentiation of nucellar cells other than the MMC. A phenotype similar to *ipt9* knockout mutants was verified in Arabidopsis overexpressing *AtGID1a.* [13]. This gibberellin receptor gene, acts on the signal transduction pathway of gibberellin in Arabidopsis [47]. In Arabidopsis, functional megaspore specification is dependent on CK signaling in the surrounding cells, with a CK-dependent pathway involved in the sporophytic-gametophytic tissue communication [22, 33]. An imbalance in the expression of *AtIPT9* and *AtGID1a*, might lead to the differentiation of additional MMC-like cells in the nucellus. In both cases, in ovules with extra MMC-like cells, only one MMC accomplished the meiotic division, only one functional megaspore and only one ES were observed, leading to infer that additional MMC-like cells do not enter gametogenesis. These developmental alterations did not interfere in the formation of viable and fertile seeds. The *AtGID1a* overexpression and *AtIPT9* knockout involved, respectively, in the pathway of gibberellin and CK synthesis bring a first impression on the possible effect of these hormones on differentiation of nucellar cells.

This work shows identification of a gene from the IPT family in sexual and apomictic *B. brizantha*, *BbrizIPT9*. *BbrizIPT9* expression suggests a role of this gene on the reproductive development of *B. brizantha*, been associated with onset of megasporogenesis in apomictic and sexual plants. The expression of a *BbrizIPT9*, localized in male and female sporocytes, and lower in apomicts than in sexuals, and effect of *IPT9* knockout in Arabidopsis, suggest involvement of *IPT9* in early ovule development.

## Supplementary Information

Below is the link to the electronic supplementary material.

Supplementary file1 (PDF 557 KB)

## Data Availability

The *Brachiaria brizantha IPT9* sequences analysed in this study were deposited in the NCBI GenBank database under the accession number MZ724659 and MZ724660. All data generated or analysed during this study are included in this published article (and its supplementary information files).
