# Supplementary material for: Identification of IPT9 in Brachiaria brizantha (syn. Urochloa brizantha) and expression analyses during ovule development in sexual and apomictic plants
Source: Mol Biol Rep. 2023 Apr 18;50(6):4887–97. doi: 10.1007/s11033-023-08295-7 (PMC10209240; doi:10.1007/s11033-023-08295-7)
Supplement: Supplementary file 1 — Supplementary file1 (PDF 557 KB) [file 11033_2023_8295_MOESM1_ESM.pdf]

Title:

Identification of *IPT9* in *Brachiaria brizantha* (*syn. Urochloa brizantha*) and expression analyses during ovule development in sexual and apomictic plants

Journal name: Molecular Biology Reports

Author names and affiliations:

Luciana Gomes Ferreira[1,2] (biolgf@yahoo.com.br); Diva Maria de Alencar Dusi[2]; (diva.dusi@embrapa.br); André Southernman Teixeira Irsigler[2] (andre.irsigler@embrapa.br); Ana Cristina Meneses Mendes Gomes[2] (ana.gomes@embrapa.br); Lilian Hasegawa Florentino[2] (lilian.florentino@embrapa.br); Marta Adelina Mendes[3] (martamendes86@gmail.com); Lucia Colombo[3] (lucia.colombo@unimi.it); Vera Tavares de Campos Carneiro[1,2] (vera.carneiro@embrapa.br).

[1]University of Brasília - UnB, Department of Biology. Campus Darcy Ribeiro S/N – Asa Norte, Brasília – DF, 70.910-900, Brazil.

[2]Embrapa Genetic Resources and Biotechnology, Parque Estação Biológica, PqEB Av. W5 Norte. Caixa Postal 02372, 70.770-917 Brasília – DF, Brazil.

[3]Università degli Studi di Milano, Dipartimento di Bioscienze, Via Celoria 26, 20133 Milan, Italy.

Corresponding author:

Vera Tavares de Campos Carneiro.

Embrapa Genetic Resources and Biotechnology, Parque Estação Biológica, PqEB Av. W5 Norte. Caixa Postal 02372, 70.770-917 Brasília – DF, Brazil. vera.carneiro@embrapa.br, +55 613448-4668.

Electronic supplementary material

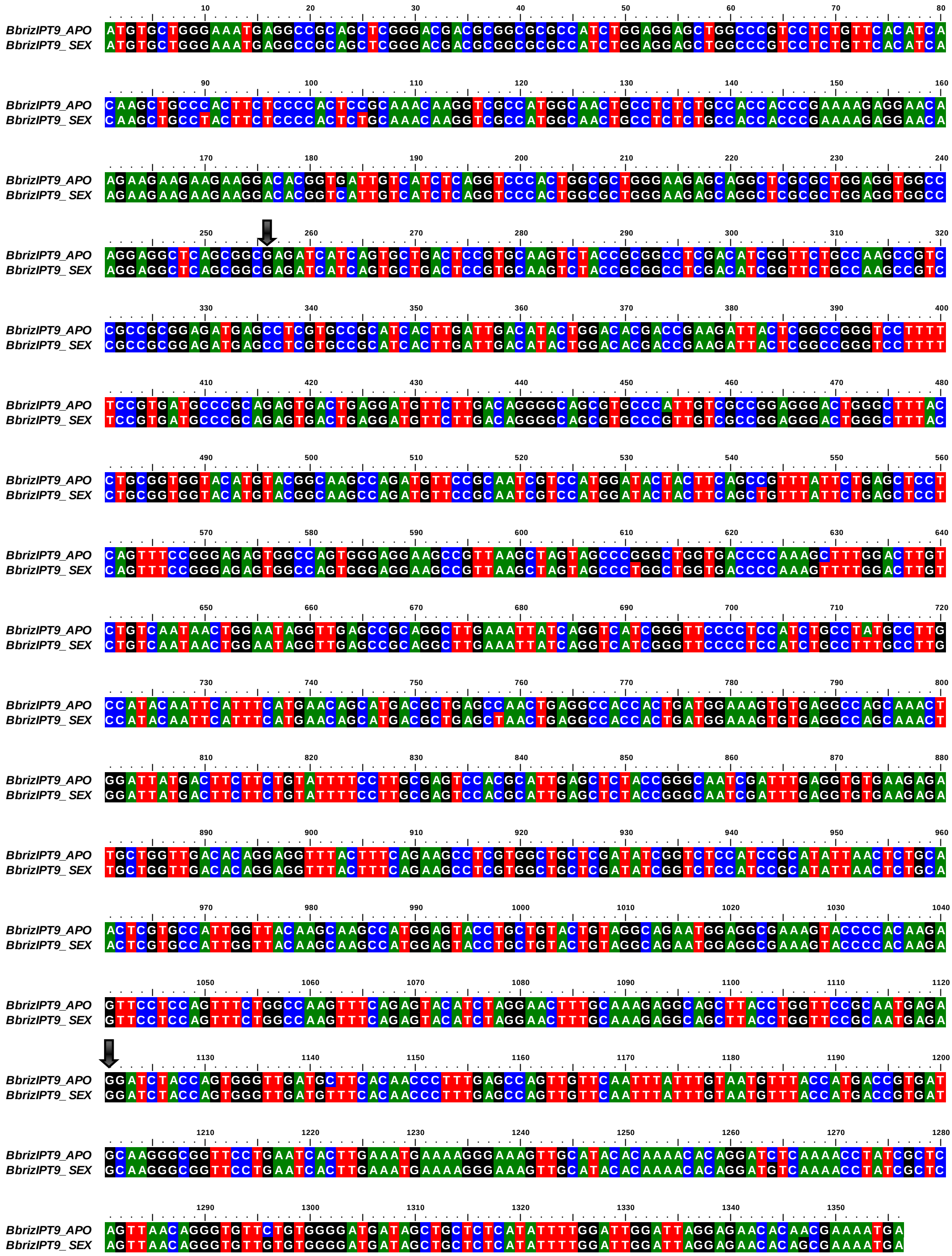

Fig. S1 Pairwise sequence alignment, by ClustalW, of *BbrizIPT9* genomic sequence from apomictic (APO) and sexual (SEX) plants. The arrows indicate the starting and the end of the region used as probe in the Southern blot analyses and in situ hybridization. In this gene the presence of introns was not detected.

```
                                                          60
BbrizIPT9_APO  MCWEMRPQLGTTRRAIWRSWPVLCSHHKLPTSPHSANKVAMATASLPPPEKRNKKKKKDT
BbrizIPT9_SEX  MCWEMRPQLGTTRRAIWRSWPVLCSHHKLPTSPHSANKVAMATASLPPPEKRNKKKKKDT

                                                         120
BbrizIPT9_APO  VIVISGPTGAGKSRLALEVARRLSGEIISADSVQVYRGLDIGSAKPSAAEMSLVPHHLID
BbrizIPT9_SEX  VIVISGPTGAGKSRLALEVARRLSGEIISADSVQVYRGLDIGSAKPSAAEMSLVPHHLID

                                                         180
BbrizIPT9_APO  ILDTTEDYSAGSFFRDARRVTEDVLDRGSVPIVAGGTGLYLRWYMYGKPDVPQSSMDTTS
BbrizIPT9_SEX  ILDTTEDYSAGSFFRDARRVTEDVLDRGSVPVVAGGTGLYLRWYMYGKPDVPQSSMDTTS

                                                         240
BbrizIPT9_APO  AVYSELLSFRESGQWEEAVKLVARAGDPKALDLSVNNWNRLSRRLEIIRSSGSPPSAYAL
BbrizIPT9_SEX  AVYSELLSFRESGQWEEAVKLVALAGDPKVLDLSVNNWNRLSRRLEIIRSSGSPPSAFAL

                                                         300
BbrizIPT9_APO  PYNSFHEQHDAEPTEATTDGKCEASKLDYDFFCIFLASPRIELYRAIDLRCEEMLVDTGG
BbrizIPT9_SEX  PYNSFHEQHDAELTEATTDGKCEASKLDYDFFCIFLASPRIELYRAIDLRCEEMLVDTGG

                                                         360
BbrizIPT9_APO  LLSEASWLLDIGLHPHINSATRAIGYKQAMEYLLYCRQNGGESTPQEFLQFLAKFQSTSR
BbrizIPT9_SEX  LLSEASWLLDIGLHPHINSATRAIGYKQAMEYLLYCRQNGGESTPQEFLQFLAKFQSTSR

                                                         420
BbrizIPT9_APO  NFAKRQLTWFRNERIYQWVDASQPFEPVVQFICNVYHDRDARAVPESLEMKRESCIHKTQ
BbrizIPT9_SEX  NFAKRQLTWFRNERIYQWVDVSQPFEPVVQFICNVYHDRDARAVPESLEMKRESCIHKTQ

                                 450
BbrizIPT9_APO  DLKTYRSVNRVFCGDDSCSHILDWIRRTQRK
BbrizIPT9_SEX  DVKTYRSVNRVLCGDDSCSHILDWIRRTQRK
```

Fig. S2 Comparison of predicted amino acid sequences of *BbrizIPT9* from apomictic (APO) and sexual (SEX) plants. The sequence of the genes obtained in the genomic database showed few differences of amino acids between apomictic and sexual genotypes (arrows). Numbers indicate the position from the start codon. The ClustalW Multiple Alignment program was used for alignment.

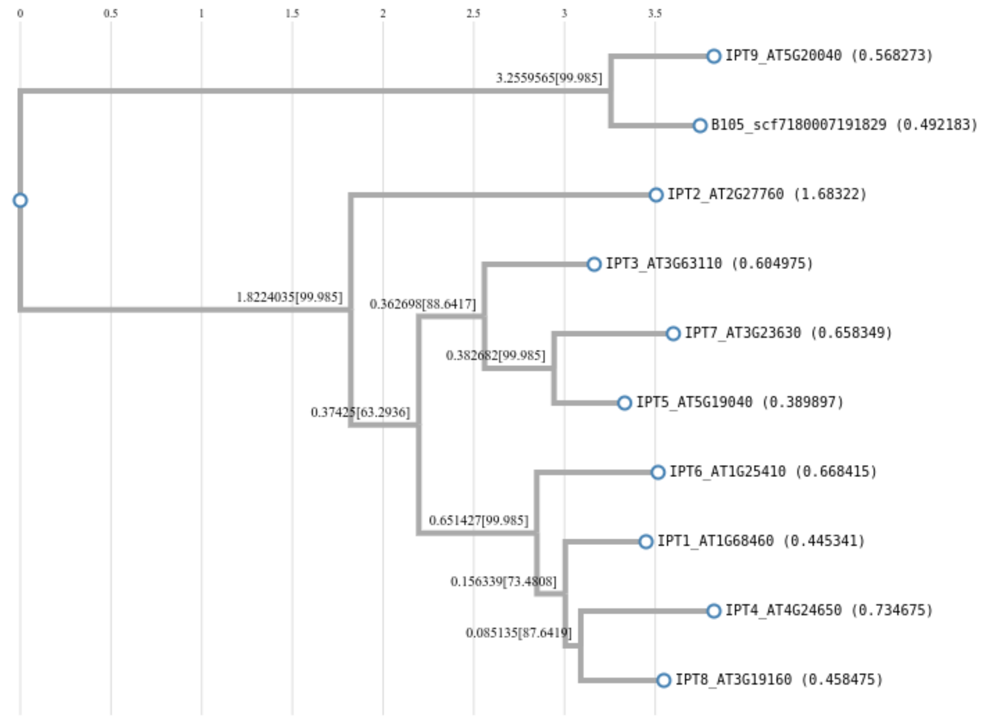

Fig.S3 Phylogenetic tree based on full protein sequences of the isopentenyltransferase (IPT) family in *Arabidopsis* and a IPT from *Brachiaria brizantha*. Construction of the phylogenetic tree was performed using the Maximum Likelihood-based method with PhyML v20160115 [39], based on 100 bootstrapped trees. Abbreviations: AT, *Arabidopsis thaliana*; B105, *B. brizantha* sexual
